# Clinical analysis of acute poisoning in children

**DOI:** 10.1186/s12887-024-04697-z

**Published:** 2024-03-25

**Authors:** Huajun Zhang, Qin Huo, Rui Jing, Meng Dong

**Affiliations:** 1 Department of Pediatrics, Yantai Hospital of Traditional Chinese Medicine, 39#, Xingfu Road, Yantai, Shandong 264000 China; 2 Department of General Medicine, The Fourth People’s Hospital Of Jinan, 50#, Shifan Road, Jinan, 250013 China; 3 Department of Pediatrics, Weifang People’s Hospital, 151#, Guangwen Road, Weifang, Kuiwen District 261000 China; 4 Department of Pediatrics, Qilu Hospital of Shandong University, 107#, Wenhuaxi Road, Jinan, Shandong 250012 China

**Keywords:** Acute poisoning, Hospitalized children, Type of poisoning, Treatment, Clinical analysis

## Abstract

**Objective:**

The clinical characteristics of hospitalized children with acute poisoning were analyzed to provide a reference for preventing poisoning and seeking effective prevention and treatment.

**Methods:**

The clinical data of 112 children with acute poisoning admitted to Qilu Hospital of Shandong University from January 1, 2018, to December 31, 2021, were collected and analyzed from different perspectives.

**Results:**

The majority of acute poisoning cases that occurred in children were in early childhood and preschool age (89 cases, accounting for 79.4%). The most common types of poisoning were pesticide poisoning and drug poisoning, and the main ways of poisoning were accidental administration via the digestive tract and accidental ingestion. Poisoning occurred slightly more in spring and summer all year round, and most children had a good prognosis after timely treatment.

**Conclusion:**

Acute poisoning often occurs in children. Parental education and intensified child supervision are needed to prevent the incidence of unintentional poisoning.

## Introduction

Acute poisoning is one of the most common emergency medical events in children, and it is also one of the important causes of accidental injuries to children. Because of the acute onset, rapid change and development of the disease, and serious harm to children’s health. It can bring a heavy burden to children, families, and even society. The World Health Organization (WHO) reported that poisoning is one of the top five causes of accidental injuries to children [1]. Poisoning in children is related to the surrounding environment, mainly acute poisoning [2]. At present, there lack of large-scale, multicenter epidemiological data research in China. This study performed a retrospective analysis of the clinical data from 112 hospitalized children admitted to the Department of Pediatrics of Qilu Hospital of Shandong University from January 1, 2018, to December 31, 2021. The major purpose is to explore possible risk factors and preventive measures to guide the clinical treatment of children with poisoning.

## Methods

### Data and methods

#### Data Sources

 All cases in this study were collected from hospitalized children admitted to the Pediatric Department of Qilu Hospital from January 1, 2018, to December 31, 2021.

#### Methods

This study was a cross-sectional study. A total of 112 hospitalized children were selected by using the case screening of Qilu Hospital of Shandong University to input “acute poisoning”. The clinical retrospective analysis was carried out according to age, sex, season, type of poison, poisoning time, visiting time, poisoning route, poisoning manifestation, injured organ, treatment method and so on.

##### Inclusion criteria

Children aged 0–18 years, discharge records to the first diagnosis as the main diagnosis of this study, and disease diagnosis following the International Classification of Diseases standard coding ICD-10 code [3] (To facilitate the classification of toxic substances). Diagnostic criteria: a clear history of poisoning by mistake or other means, can find contact with poison or container; toxicants and specific enzymatic changes could be detected in the blood and urine of some children.

##### Grouping method

According to the growth and development stage of children in pediatrics, it is divided into neonatal and infant periods (0–1 year), early childhood (1–3 years), preschool periods (3–7 years), school age and adolescence (≥ 7 years). Divided by gender.

According to China’s climatic and astronomical seasons, it is divided into spring (March-May), summer (June-August), autumn (September-November), and winter (December-February).

Classification and Causes of Poisoning In this study According to ICD-10 code T36.051-T65.951, the toxic substances are divided into drug poisoning (including Chinese herbal medicine), chemical poisoning, pesticide poisoning, rodenticide poisoning, carbon monoxide (CO) poisoning, and other substances [3]. According to the code classification of carbon monoxide poisoning as a harmful gas poisoning category, this classification is listed separately. The causes of poisoning are divided into accidental poisoning (refers to children in life due to contact, aspiration, ingestion, and other ways of contact with poisons) and intentional poisoning (refers to the poisoning caused by child’s intentional medications for various reasons and intentional feedings of others).

Clinical manifestations and laboratory tests in children are following medical records. Clinical manifestations include the digestive system, respiratory system, blood circulation system, nervous system, and patients without clinical symptoms. Laboratory tests included liver function, renal function, myocardial enzymes, cholinesterase, coagulation system, and other tests. According to laboratory results, they were divided into groups with or without organ damage.

#### Statistical analysis

SPSS 25.0 statistical software was used to process the data. The distribution of general count data, such as age, sex, and toxicant species, was described by the number of cases and composition ratio (%). The x2 test or statistical inference was used to compare the categorical variable components, and the test was used to compare the count variable groups. *P* < 0.05 was considered statistically significant.

## Results

### Gender and age distribution of children

**Fig. 1:**
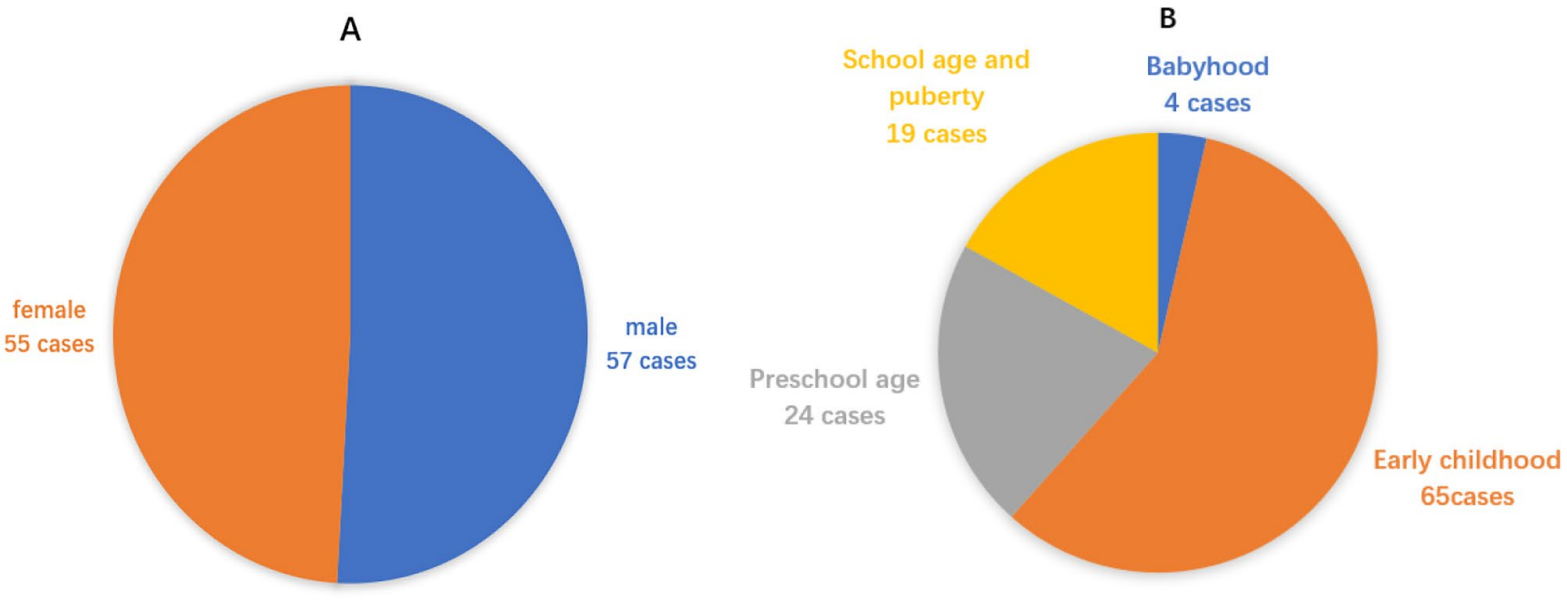
**A** Gender distribution of poisoning in children. **B** Age distribution of poisoning in children. According to the age of the children, they were divided into 4 groups. Less than 1 year old was divided into the neonatal and infant groups, 1 to 3 years old was divided into the early childhood group, 3 to 7 years old was divided into the preschool group, and 7–18 years old was divided into the school age and puberty groups

Among 112 children hospitalized for acute poisoning, 57 (50.9%) were male and 55 (48.9%) were female (Fig. 1A), with a male-to-female ratio of 1.04:1. Among them, there were 4 cases (3.6%) in the neonatal and infant group, 65 cases (58.0%) in the early childhood group, 24 cases (21.4%) in the preschool group, and 19 cases (17.0%) in the school-age group and the puberty group (Fig. 1B). The proportion of early childhood was the highest, and the difference was statistically significant (*P* < 0.05).

### Time distribution of poisoning in children

Among children with poisoning, there were 23 cases (20.5%) in 2018, 28 cases (25.0%) in 2019, 27 cases (24.1%) in 2020, and 34 cases (30.4%) in 2021. There were 34 cases (30.4%) in spring, 40 cases (35.7%) in summer, 15 cases (13.4%) in autumn, and 23 cases (20.5%) in winter. The proportions in spring and summer were close to that in autumn, and the difference was not statistically significant (*P* > 0.05) (Table 1). There were 7 cases in January, 4 cases in February, 14 cases in March, 10 cases in April, 10 cases in May, 13 cases in June, 15 cases in July, 12 cases in August, 5 cases in September, 6 cases in October, 4 cases in November, and 12 cases in December. Most cases were in July, followed by March, and the fewest were in February and November, with 4 cases each.

**Table 1:** Time distribution of poisoning in children

Year	Patients	Spring	Summer	Autumn	Winter	X^2^	*P*
2021	34	13	7	6	8	14.851	0.123
		38.2%	20.6%	17.6%	23.5%		
2020	27	5	13	2	7		
		18.5%	48.1%	7.4%	25.9%		
2019	28	6	12	3	7		
		21.4%	42.9%	10.7%	25.0%		
2018	23	10	8	4	1		
		43.5%	34.8%	17.4%	4.3%		

### Causes and approaches of poisoning

Among the 112 hospitalized children, 102 cases were accidentally poisoned, 10 cases were intentionally poisoned, of which 92 cases (82.1%) were accidentally poisoned by ingestion, 6 cases (5.4%) were accidentally poisoned by aspiration, 2 cases (1.8%) were accidentally exposed by parents, 1 case (0.9%) was caused by personal contact, and 1 case (0.9%) was poisoned by medication. Among intentional poisoning, 8 cases (7.1%) were self-administered, and 2 cases (1.8%) were forcibly administered. There were 103 cases (91.9%) with digestive system ingestion, 6 cases (5.4%) with respiratory system ingestion, and 3 cases (2.7%) with skin contact in all cases. and a detailed description is shown in Table 2. Among all of the cases, seven occurred outdoors (including factories or farmland), and the rest occurred at home.

**Table 2:** Comparison of causes of acute poisoning in children with different characteristics of acute poisoning

group		cases	mis-using	mis-eating	self-administered	inhalation	contact	mandatory	others	X^2^	*P*
sex	male	57	41	7	3	2	1	2	0	3.438	0.695
			73.2%	12.5%	5.4%	3.6%	1.8%	3.6%	0.0%		
	female	55	37	7	5	4	2	0	0		
			67.3%	12.7%	9.1%	7.3%	3.6%	0.0%	0.0%		
age	infant	4	3	0	0	1	0	0	0	57.525	0.00
			75.0%	0.0%	0.0%	25.0%	0.0%	0.0%	0.0%		
	young child	65	56	7	0	0	1	1	0		
			86.2%	10.8%	0.0%	0.0%	1.5%	1.5%	0.0%		
	preschool	24	15	5	0	2	1	1	0		
			62.5%	20.8%	0.0%	8.3%	4.2%	4.2%	0.0%		
	school age and adolescent	19	4	2	8	3	1	0	1		
			21.1%	10.5%	42.1%	15.8%	5.3%	0.0%	5.3%		
season	spring	34	27	2	5	0	0	0	0	4.508	0.029
			79.4%	5.9%	14.7%	0.0%	0.0%	0.0%	0.0%		
	summer	40	27	5	0	2	3	2	1		
			67.5%	12.5%	0.0%	5.0%	7.5%	5.0%	2.5%		
	autumn	15	10	4	1	0	0	0	0		
			66.7%	26.7%	6.7%	0.0%	0.0%	0.0%	0.0%		
	winter	23	14	3	2	4	0	0	0		
			60.9%	13.0%	8.7%	17.4%	0.0%	0.0%	0.0%		

#### Types of poisoning

The types of poisoning in children were pesticide poisoning, drug poisoning, rodenticide poisoning, chemical drug poisoning, and other poisoning. There were 43 cases (38.4%) of pesticide poisoning, 31 cases (27.7%) of drug poisoning, 14 cases (12.5%) of rodenticide poisoning, 12 cases (10.7%) of chemical drug poisoning, 9 cases (8.0%) of other poisoning, and 3 cases (2.7%) of poisoning. The top three were pesticides, drugs, and rodenticides (Table 3). In terms of gender, there were 23 cases of pesticide poisoning, 12 cases of drug poisoning, 10 cases of rodenticide poisoning, 6 cases of chemical poisoning, 5 cases of other poisoning, and 1 case of CO poisoning in hospitalized male children. As for female hospitalized children, there were 20 cases of pesticide poisoning, 19 cases of drug poisoning, 6 cases of chemical poisoning, 4 cases of rodenticide poisoning, 4 cases of other poisoning, and 2 cases of CO poisoning. This study found that the order of the top three poisoning types of boys and girls was different. The top two were pesticide and drug poisoning, with no significant statistical difference.

**Table 3:** Types of poisoning in children of different age groups

group		cases	drug	pesticide	rodenticide	chemical drug	CO	others	X^2^	*P*
Age	infant	4	1	2	0	1	0	0	22.273	0.039
			25.0%	50.0%	0.0%	25.0%	0.0%	0.0%		
	young child	65	25	18	8	6	8	0		
			38.5%	27.7%	12.3%	9.2%	12.3%	0.0%		
	preschool	24	10	4	5	5	0	0		
			41.7%	16.7%	20.8%	20.8%	0.0%	0.0%		
	School age and adolescent	19	7	7	1	0	1	3		
			36.8%	36.8%	5.3%	0.0%	5.3%	15.8%		

### Poisoning treatment time and treatment

Except for 1 case that could not seek medical treatment in time because of kidnapping and a few children did not dare or were unwilling to tell their parents, most of them went to the emergency department of the hospital immediately. The median consultation time was 6 h, indicating that most parents saw a doctor within 24 h of the poisoning. The median duration of hospitalization was 6 days, with a mean of 6.50 ± 4.782 days (range 1–36 days). Seventeen cases (15.2%) resulted in hospitalization not more than 2 days, including 10 cases of automatic discharge. There were 54 cases (48.2%) with hospital stays ranging from 3 to 7 days, 37 cases (33.0%) lasting 7–14 days, and 4 cases (3.6%) exceeding 14 days. Only 1 case was hospitalized for over 30 days, with half of them being visible. In addition, treatment times varied for different types of poisoning: pesticide poisoning averaged 7.19 ± 6.280 days, drug poisoning 6.19 ± 4.167 days, rodenticide poisoning 5.36 ± 2.274 days, chemical poisoning 6.25 ± 3.745 days, other poisoning treatment time 6.00 ± 3.279 days, and CO poisoning treatment time 7.67 ± 1.528 days. However, the difference in poisoning hospitalization days was not statistically significant.

### Clinical characteristics and laboratory examination of poisoning

In this study, the primary treatment for children was mainly on the digestive system, followed by asymptomatic symptoms, nervous system, blood circulatory system, and respiratory system symptoms. Most children had two or more clinical manifestations simultaneously, such as vomiting and abdominal pain in the digestive system or the simultaneous presence of vomiting, convulsion, and cough. When it comes to clinical symptoms, vomiting (37 cases), abdominal pain (10 cases), nausea (6 cases), and diarrhea (3 cases) were the main digestive system manifestations. The nervous system exhibited limb convulsions (11 cases, with 2 cases of convulsive seizures), lethargy (7 cases), dizziness (6 cases), confusion (3 cases), and headache (3 cases). The circulatory system included irritability (7 cases, with 2 cases showing excitement at the same time, all due to sibutramine poisoning), limb weakness (7 cases, of which 3 cases were significant overall weakness), and numbness of the lower extremities (1 case). The main manifestations of the respiratory system were cough (3 cases), dyspnea (1 case), and positive three-notch sign (1 case). Other manifestations were mainly perioral ulcers, red lips and swelling, tongue numbness, consciousness of bitter mouth, and purpura. Overall, gastrointestinal and nervous system injuries were predominant, with symptoms like vomiting, abdominal pain, convulsions, lethargy, dizziness, etc. (Table 4). Laboratory examination results showed multiple organ damage in most cases, among which pesticide poisoning causes the most damage. Myocardial damage accounts for the majority, manifested as weakness of limbs, sweating, poor spirit, irritability, etc. However, about 29 cases (25.9%) showed no obvious abnormalities in laboratory examination (Table 5). After hospitalization, all children showed improvement in blood routine, liver function, kidney function, myocardial enzyme, coagulation series, electrolytes, etc. Blood cholinesterase was detected for pesticide poisoning, and neuron-specific enolase (NSE) was added for conscious changes or unexplained poisoning. Toxicant testing was performed conditionally.

**Table 4:** Clinical manifestations of different poisons

types of poison	cases	digestive	asymptomatic	nervous	circulation	breathing	others
pesticide	57	20	21	7	5	1	3
drug	46	6	10	17	8	4	1
rodenticide	16	5	7	1	2	1	0
chemical drug	16	10	1	3	1	0	1
others	14	7	1	3	1	2	0
CO	3	0	0	3	0	0	0

**Table 5:** Laboratory examination of different toxic abnormalities

types of poison	cases	myocardium	liver	kidney	NSE	electrolyte	blood	cholinesterase	other	no abnormal
pesticide	70	22	5	4	2	6	7	9	4	11
drug	42	13	4	2	6	2	2	0	3	10
rodenticide	19	8	2	1	1	0	2	1	0	4
chemical drug	18	7	1	1	0	3	2	1	1	2
others	13	6	2	0	1	1	1	0	0	2
CO	5	2	1	1	0	0	0	0	0	1

### Treatment and outcome

Among the children with poisoning, 57 (50.9%) were treated with gastric lavage, and 24 (21.4%) were treated with special drugs (such as atropine, acetamide, sodium thiosulfide, and methylene blue). All organophosphorus poisoning cases were treated with atropine combined with praloxime, and 15 (13.4%) were treated with blood purification technology. Most of the poisonings have no specific drug treatment, and gastric lavage treatment accounts for the vast majority. In 2021, there were 3 cases of poisoning from illegally added sibutramine, disguised as harmless chocolate, and 1 case of parents taking the initiative to their children, resulting in palpitations, convulsions, and coma. After initial gastric lavage, all patients recovered after applying hemoperfusion in the ICU. At present, blood purification technology is increasingly important in treating poisoned children. In the discharge statistics, 97 patients were cured and discharged, 14 patients were discharged automatically, and unfortunately, 1 patient succumbed to multiple organ failure.

## Discussion

Poisoning means that the poison enters the human body through the skin, mucous membrane, respiratory tract, digestive tract, and other ways in a short time, causing damage to the body and organ dysfunction. With the development of the Chinese social and economic level and the acceleration of industrialization, an increasing number of poisons can be exposed to children in daily life. Accidental injuries have become an important cause of casualties in children and adolescents. Acute poisoning is the most common pediatric emergency. This study found that the average age of hospitalized children with poisoning was 4.24 ± 3.91 years old, with median of 2.6 years old. The age range from one month and 17 days to 16 years old, with a large age span. Poisoning children were more common in early childhood and preschool age, accounting for approximately 79.5% of the total number of hospitalizations, which was basically consistent with domestic research [4]. Combined with the physiological development stage of children, children’s intelligence gradually develops at this stage. They can walk independently, with an increased range of activities, strong curiosity, and imitation skills, yet possess a limited awareness of risk, making them particularly exposed to poisoning incidents. Interestingly, this study revealed minimal variance in poisoning cases between genders, which was different from other studies in China. In the past, it was considered that boys were more prone to poisoning because of their liveliness, and boys were more favored than girls. However, according to the data of the seventh population census of Shandong Province, the sex ratio of the population born in 2021 was 111.95, which was 7.45 lower than that in 2010, and 0.65 higher than the national average. The continuous improvement of the gender structure of the population in the province led to a small difference in gender differences in poisoning. Additionally, the relatively small sample size might have impacted the observed gender patterns [5].

This study found that the top four types of children’s poisoning are pesticide poisoning, drug poisoning, rodenticide poisoning, and chemical poisoning, which is not very consistent with the analysis of children’s poisoning at home and abroad [6–8]. Pesticide poisoning has occurred relatively frequently for some time, as shown by previously published papers from different countries [9–12]. The incidence of this type of poisoning varies geographically and historically [13]. Several studies conducted in India, Sri Lanka, and Bangladesh showed a pesticide-dominant poisoning pattern, with organophosphates being the most common pesticide [14–16]. With the gradual improvement of the rural contract responsibility system for linked production after the reform and opening up, rural areas in Shandong have also begun to implement a variety of operations. The use and storage of various pesticides are controlled by farmers’ purchase, and the pesticides are stored near food or drinks without following the storage requirements, resulting in exposure and poisoning of children. This is also consistent with our statistics.

Pesticides and rodenticides have always been common in rural areas, and since the reform and opening up, it has been a common social problem. With the acceleration of urbanization in Shandong Province in recent years (According to the website of Shandong Provincial Bureau of Statistics, the urbanization rate of the province’s permanent resident population increased from 52.03 to 63.94% by the end of 2012 to 2021. In 2002, the urbanization rate was only 29%), new problems reappeared. The boundaries between rural and urban areas have become increasingly blurred as rural residents migrate to cities. For example, children born in rural areas but raised in the city contribute to statistical confusion. This study is performed based on the statistics of nearly 4 years from 2018 to 2021, and the phenomenon is particularly evident. Usually, children live with their parents in the cities but may visit their elders in the countryside during weekends and holidays. Many elderly people in rural areas store pesticides, rat poison, and drugs indiscriminately, making them easily accessible to children and increasing the risk of accidental poisoning. Therefore, this study cannot perform the classification statistics of urban and rural areas from the statistical aspect. Additionally, statistics show that pesticide and rat poisonings are decreasing, while drug and chemical poisonings are increasing. This shift is particularly evident in the newly emerged poisoning of children’s diet pills, a novel problem linked to urbanization. After settling down in cities, they are more affected by the fashion trend. Children, especially adolescent girls, mistakenly take diet pills. But the banned drugs, like sibutramine, were added to these pills, causing drug poisoning.

Sibutramine is a class of highly effective drugs for the treatment of obesity by disrupting the regulatory function of hormones in the body, stimulating sympathetic nerve, inhibiting appetite, and achieving weight loss effect. However, Sibutramine cardiovascular outcome (SCOUT) trial showed that it has adverse effects on human cardiovascular disease, especially in people with heart disease. The FDA and China’s National Drug Administration withdrew Sibutramine from the market. Despite this, unscrupulous vendors have illegally added Sibutramine to health foods, leading to the existence of Sibutramine over-the-counter weight loss formulas on the market. Clinical evidences confirmed that it has great harm to the heart and nervous system and even leads to the risk of serious cardiovascular diseases, such as non-fatal heart attack and non-fatal stroke, which seriously affects human health [17–19]. However, there are few studies on it after its prohibition, and it is only symptomatic treatment without specific treatment, and the pediatric intensive care unit of our hospital adopts hemoperfusion, plasma exchange, and other treatments.

Although complete statistics on the difference between urban and rural poisoning are lacking, there is a noticeable trend that pesticide and rodent poisoning occurs in rural areas, which is consistent with other reports in China [20]. Because rural areas need farming, coupled with the living environment, as well as adverse supervision, a large number of pesticides and rodenticides can be purchased. In addition, the awareness of garbage disposal is inadequate, so it is easy for children to contact and take it by mistake. Moreover, the spraying time of pesticides is different in every family in the countryside, which easily causes children to spray pesticide fruits by mistake when playing in the wild. Furthermore, spraying pesticide seeds and preparing food with rodenticides easily cause children to eat by mistake due to negligence and forgetting.

The main way of poisoning is the digestive tract, mistaking or eating by mistake, mostly occurring in children and school-age children. Conversely, adolescents are mostly self-administered, aligning with current domestic and international statistics [21, 22]. This trend is in line with the characteristics of different stages of children’s growth and development. Children exhibit high levels of curiosity and imitation, coupled with a limited awareness of danger. The problem of adolescents, because of their poor psychological situation, is also related to their current education and social environment. Parents’ scolding, laughing among classmates, and teachers’ punishment easily make adolescents rebellious and world-weary. In addition, mental illness is also an accidental risk factor for adolescents.

In this study, the median time of children’s visit to our hospital was 16 h. However, this cannot fully represent the parents of the treatment period. Because it is the date of treatment of hospitalized children, not the date of emergency addimition. Many rural children go to two or even three hospitals before coming to the best Qilu hospital in Shandong Province (Shandong University Qilu Hospital is a third-level A general hospital entrusted (managed) by the National Health Commission, and is the first batch of national regional medical centers built by the CPC and the province to lead and the main construction unit, and the national regional medical center construction output hospital. Pediatrics is a key discipline of medicine and health in Shandong Province. The pediatric inpatient Department includes 8 specialized wards with 246 inpatient beds. The department has 280 medical and technical staff. There are 66 physicians and 214 nursing and technical staff. Ranked first in Shandong Province in the Fudan Edition of the “China Hospital Comprehensive Ranking 2022”.). On average, children stayed in the hospital for 6.5 days. The duration of hospitalization is related to the type of poisoning, the time of the poisoning, the severity of the poisoning, and the financial burden on parents. Most children showed improvement after treatment. Generally, mistakenly taking drugs leads to lighter clinical symptoms than taking poisons themselves. Because children are unintentionally poisoned, the poisoning is single, and the amount is even small. Self-administration includes self-injury, and the dose is generally large, especially when he (or she) wishes to commit suicide by taking poisons.

Due to the different types and different doses in acute poisoning, the clinical manifestations are different, showing complexity. Some cases may even be asymptomatic. Most toxins cause organ damage or even multiple organ damage, but mainly in the digestive system and nervous system. Pesticides, rodenticides, and chemical poisoning have been more common in the digestive system, causing nausea, vomiting, and abdominal pain. Drugs and CO poisoning were mainly in the nervous system, resulting in confusion, lethargy, irritability, convulsions, etc. For laboratory tests, some children show normal results, but half of the children have myocardial enzyme damage. Cholinesterase abnormalities appear in pesticide poisoning (81.8%). NSE abnormalities mainly appear in drug poisoning. NSE is also a specific and sensitive biomarker to judge the degree of craniocerebral injury and disease prognosis. Notably, pesticide poisoning caused the most abnormal indicators.

## Deficiencies and prevention

The research period of this study is short, with a small number of cases, focusing on hospitalized children. This limited scope may not fully reflect the clinical characteristics, especially the clinical manifestations. Emergency physicians, especially those on ambulance duty, are likely to see the earliest manifestations of children, potentially affecting the time and performance of visits. Additionally, the area is relatively single, not a multicenter study. It is impossible to compare the acute poisoning data of other hospitals in Jinan horizontally and then to compare the data of prefecture-level hospitals and county-level hospitals vertically, which cannot capture the medical characteristics of Shandong. Furthermore, the Hospital Information Systems (HIS) systems of hospitals in various cities are different and cannot be unified. Therefore, the statistical data are local statistics, and may not accurately reflect broader trends.

China is geographically divided into the north and south by the “Qinling Mountains-Huaihe River”, leading to distinct environmental differences. The causes of poisoning are also different. In the south, special animals and plant poisoning such as mushroom poisoning and snake bite poisoning are highly prevalent. Conversely, in the northern region of Shandong, the poisoning is caused by drugs, pesticides, rat poison, and chemicals. This also causes differences in poisoning statistics when reviewing papers, especially since the statistics of multi-center studies vary widely across the country and in some regions.

This study only differentiated between cured and improved discharge, automatic discharge, death, etc., without categorizing it into cure, improvement, ineffectiveness, etc. Because it mainly focused on statistical observation, without follow-up tracking, assessing factors such as different types of poison, doses, children’s growth, development, and psychological impact isn’t possible. We only make statistics based on the diagnosis on the first page of the medical record. To date, there are few large-scale multicenter follow-up studies, and we urge attention to this area from the country.

With the acceleration of Chinese economic development and urbanization process, coupled with policy changes, earth-shaking changes have taken place in rural areas at present. As more rural population migrate to the city, the number of left-behind elderly and children has decreased. Moreover, the rural areas are merging into villages, and the urban areas are being integrated. Consequently, the traditional boundaries between rural and urban zones have become increasingly blurred, making it challenging to accurately delineate between them. In particular, the retrospective study span lengthy periods, resulting in inconsistencies in medical record. Children will be more likely to find poisoning and other accidental injuries.

Prevention of acute poisoning is always more important than treatment. According to the different types of poisoning and their epidemiological characteristics, the following suggestions are proposed: (1) the drug must be stored out children’s sight and reach, or equipped with a special storage locked box; (2) commonly used pesticides should have prominent warning signs, and the banned pesticide in industry and commerce departments must increase oversight; (3) stringent control measures are necessary for drugs, especially the static anesthesia drugs. Prescription drugs should only be sold with a doctor’s prescription, and the drug supervision department should strengthen market supervision; (4) parents and schools should pay more attention to the mental health of young people, rather than solely focusing on academic performance; (5) primary hospitals and health centers should publicize at the grassroots level during the peak farming seasons, including educating parents to strengthen care and basic rescue measures. Furthermore, farmers should be informed about pesticide management regulations when purchasing, and medical personnel should educate other individuals traveling to rural areas.

In terms of prevention, having proficient first-aid skills is essential for rescuing children from poisoning, as children are not the epitome of adults. At present, provincial hospitals have featured poisoning departments, either as standalone units or as part of their emergency departments. However, many children’s hospitals and pediatrics in China lack dedicated children’s poisoning departments. This deficiency leads to a lack of experience in children poisoning rescue and hampers their ability for early identification.

## Data Availability

The authors confirm that the data supporting the findings of this study are available within the article.
